# Editorial: Multi-modal learning and its application for biomedical data

**DOI:** 10.3389/fmed.2023.1342374

**Published:** 2024-01-16

**Authors:** Jin Liu, Yu-Dong Zhang, Hongming Cai

**Affiliations:** ^1^School of Computer Science and Engineering, Central South University, Changsha, China; ^2^University of Leicester, Leicester, United Kingdom; ^3^South China University of Technology, Guangzhou, China

**Keywords:** multi-modal learning, biomedical data, machine learning, deep learning, precision medicine

With the rapid development of biomedical testing methods and the explosive growth of biomedical data, multimodal data can better meet the precise diagnosis of diseases, such as medical images and histological information can reflect the condition of the person more comprehensively. This provides a rare opportunity for researchers to carry out multimodal learning of biomedical data, deep mining and fusion of data, and discoveries on medical research.

Among the received articles, Asim et al. use multimodal learning to predict key miRNAs from miRNA sequences and Yan et al. improve the prediction of host-virus inter-protein interactions. These articles demonstrated the broad prospect of multimodal learning in molecular biology research. Meanwhile, the analysis of medical images also plays an important role in clinical applications. Refaee et al. differentiate between idiopathic pulmonary fibrosis (IPF) and non-IPF interstitial lung diseases (ILDs) in patients by multimodal analysis. Sato et al. use multimodal learning to improve the quality of assessment for predicting the dose range of proton therapy. Jovel and Greiner discuss the application of machine learning methods in biomedical research. These articles point to the fact that the development of multimodal learning techniques will do well in biomedical data analysis. All of these articles demonstrate the broad prospects of artificial intelligence techniques such as multimodal learning, deep learning, and machine learning in the biomedical field.

Although multimodal learning has a promising application on biomedical data, there are many challenges to face when dealing with multimodal medical datasets, such as Park et al. improve algorithmic robustness to different imaging devices through adversarial generative networks. How to explore the advantageous features of different modality data, the intrinsic correlation between different data, the over-reliance on a certain single modality data and the problems of model interpretability and robustness still need to be involved by a wide range of researchers.

In summary, these articles are an exploration of the rapidly growing field of artificial intelligence (AI) in biomedical research. These studies utilize multimodal learning techniques to exploit the wealth of information from different biomedical data sources, including molecular sequences, medical images, and clinical information. The breadth of applications, from predicting molecular interactions to improving diagnostic accuracy and treatment planning, highlights the potential of AI in healthcare.

The articles by Asim et al. and Yan et al. demonstrate the broad promise of multimodal learning in molecular biology, demonstrating its ability to predict key miRNAs and improve understanding of host-virus protein interactions. These studies help to investigate molecular complexity and provide new ways for further treatment and intervention.

Refaee et al. distinguished lung diseases through multimodal analysis. By combining medical imaging and other data from different modalities, this study helps to accurately diagnose the disease, with potential implications for intervention and treatment options.

Sato et al. used multimodal learning to improve the quality of assessment of dose range prediction for proton therapy. This study shows the potential of multimodal learning in practical applications for advances in AI-driven medicine, especially in the field of radiation oncology.

Jovel and Greiner discussed the application of machine learning methods to biomedical research in a comprehensive manner and is a valuable resource for highlighting the diversity of AI approaches. It provides many different ideas for researchers exploring the integration of AI into different biomedical fields.

However, numerous challenges remain in today's development, as Park et al. improve the robustness of algorithms to different imaging devices through adversarial generative networks. Issues that need to be addressed include modal data exploration, intrinsic relevance understanding, over-reliance on specific data modalities, and challenges related to model interpretability and robustness, highlighting the relentless effort required to fully utilize the potential of multimodal learning in biomedical data analysis.

These articles advance the field of AI in biomedical data, not only demonstrating direct applications in biomedical research, but also outlining the challenges that require continued innovation. As multimodal learning evolves, methods that consider advanced technologies and clinical applications will drive precision medicine forward. Multimodal learning will drive biomedical research to the next level.

## Author contributions

JL: Writing—original draft, Writing—review & editing. Y-DZ: Writing—review & editing. HC: Writing—review & editing.

